# The Effects of Acute High-Intensity Interval Training on Hematological Parameters in Sedentary Subjects

**DOI:** 10.3390/medsci5030015

**Published:** 2017-07-19

**Authors:** Muaz Belviranli, Nilsel Okudan, Banu Kabak

**Affiliations:** 1 Division of Sports Physiology, Department of Physiology, School of Medicine, Selcuk University, Konya 42250, Turkey; nokudan@selcuk.edu.tr; 2 Department of Health, Turkish Republic Ministry of Youth and Sports, Ankara 06820, Turkey; denizemre35@hotmail.com

**Keywords:** acute high-intensity interval training (HIIT), red blood cell, leucocyte, platelet, Wingate test

## Abstract

The objective of the study was to determine the effects of acute high-intensity interval training (HIIT) on hematological parameters in sedentary men. Ten healthy, non-smoker, and sedentary men aged between 18 and 24 years participated in the study. All subjects performed four Wingate tests with 4 min intervals between the tests. Blood samples were collected at pre-exercise, immediately after, 3 and 6 h after the fourth Wingate test. Hematological parameters were analyzed in these samples. The results showed that hematocrit percentage, hemoglobin values, red cell count, mean cell volume, platelet count, total white cell count, and counts of the white cell subgroups increased immediately after the acute HIIT and their values began to return to resting levels 3 h after exercise, and completely returned to resting levels 6 h after exercise. In conclusion, acute HIIT causes an inflammatory response in blood.

## 1. Introduction

It is generally known that both acute and chronic exercises induce several hematological changes in humans [1]. It has been ascertained that exercise-induced hematological changes are dependent on the type, intensity and duration of exercise. Additionally, several factors such as training status, gender, age, environmental conditions and nutritional status of the subjects also play an important role [1]. It has been reported that acute exercise has a significant effect on the rheological properties of the blood, and after acute exercise there is an increase in plasma viscosity and erythrocyte rigidity but a decrease in sedimentation rate [2].

Athletes who have been participating in an intense training program reported having decreased hemoglobin and hematocrit values and this situation is known as athlete’s anemia [3]. It has been generally shown that acute submaximal exercise increases the number of erythrocytes, leucocytes and platelets, hematocrit values and hemoglobin concentrations significantly as compared to pre-exercise values and these increments depend on plasma losses caused by the exercise [4]. It is asserted that short-time exercise performed until exhaustion increases the number of leukocytes and this situation cannot be explained by the hemoconcentration mechanism alone. Also, it can be associated with metabolic changes such as ischemia that occur during exercise and increased muscle activity leads to a greater incidence of capillary swelling and leukocyte adherence to venules [5]. Similarly, acute submaximal exercise increases leukocyte subgroups and it has been demonstrated that this elevation is related to the severity of exercise [6]. Studies [7,8] showed that these changes in hematological parameters occur immediately after exercise; whereas, it returns to resting levels within 24 h following exercise.

High-intensity interval training (HIIT) is defined as short, high-intensity exercise which contains low-intensity interval exercises or recovery periods [9]. During the training sessions, different forms of HIIT can be applied by changing several variables such as intensity, duration, interval number, duration of recovery, etc. [10,11,12]. An increasing amount of evidence has revealed that HIIT stimulates physiological parameters just like medium-intensity continuous training in spite of reduced exercise volume [13]. Furthermore, recent studies [14,15,16] showed that HIIT is superior to medium-intensity continuous training.

To the best of our knowledge, two recently published studies [17,18] have investigated the effects of different acute HIIT protocols on hematological parameters in overweight subjects and hypoxic conditions; however, no study to date has investigated the effects of acute HIIT on hematological parameters in different time intervals in the sedentary subjects. Since HIIT stimulates different physiological responses, in the present study, we hypothesized that acute HIIT may affect hematological parameters differently when compared to acute aerobic, anaerobic and resistance exercises. Therefore, the aim of this study was to investigate the effects of acute HIIT on hematological parameters.

## 2. Materials and Methods

### 2.1. Participants

Ten sedentary male subjects aged between 18 and 24 years participated in this study (age: 20.00 ± 1.33 years, body mass: 70.30 ± 8.68 kg, height: 173.2 ± 6.1 cm). All participants were healthy, none of them had smoking habits, use of any medication or antioxidant supplements. The sedentary subjects had a sedentary lifestyle with a distinct class of behaviors such as sitting, and watching TV characterized by little physical movement and low energy expenditure. The study was approved by Non-invasive Clinical Research Ethics Committee of Medicine Faculty of Selcuk University (Ethical code: 2015/6). Before starting the study, participants were informed about the method and potential risks of the study and informed consent was signed. Body mass and height evaluations were performed in the morning. Body mass was measured with light clothes using an electronic scale and height was measured in an upright position without shoes.

### 2.2. Exercise Tests

One-week following the familiarization session, a 30-s Wingate test was performed four times, separated by a 4-min resting period between the bouts as acute HIIT. The familiarization session included two Wingate tests separated by a four-minute resting period between the tests. The Wingate test was performed on an Ergometric 894E Peak Bike (Monark Exercise, A.B.; Varberg, Sweden) against a resistance of 75 g kg^−1^ body weight. The participants were allowed to pedal unloaded and instructed to reach a pedaling rate of 100 revolutions per minute. The predetermined load was applied to the flywheel automatically by the Monark Anaerobic Test Software and the participants were verbally encouraged to maintain as high a pedaling rate as possible throughout the 30-s test duration. It has been demonstrated that this type of exercise protocol corresponded to acute HIIT [19].

### 2.3. Blood Sampling and Hematological Analysis

On arrival, each participant was asked to lie down and a catheter was inserted into an antecubital vein. Four blood samples were collected: (1) at rest; (2) immediately after; (3) 3 h after and (4) 6 h after the exercise tests. Blood samples were collected in K3 EDTA tubes (3 mL vacutainer tube; BD Vacutainer, Franklin Lakes, NJ, USA) and stored at room temperature until measurement, which was performed in all cases within 2 h after venipuncture. Hematological parameters were analyzed using Siemens ADVIA 2120^®^ hemoautoanalyzer (Siemens, A.G.; Erlangen, Germany) according to the manufacturer’s instructions. Hematocrit (%), hemoglobin (g dL^−1^), red cell count (×10^12^ L^−1^), mean cell volume (fL), mean cell hemoglobin (pg), platelet count (×10^9^ L^−1^), white cell count (×10^9^ L^−1^), neutrophil count (×10^9^ L^−1^), Neutrophils (%), eosinophil count (×10^9^ L^−1^), eosinophils (%), basophil count (×10^9^ L^−1^), basophils (%), lymphocyte count (×10^9^ L^−1^), lymphocytes (%), monocyte count (×10^9^ L^−1^), and monocytes (%) were analyzed in these samples. The coefficient of variation (CV) values are 2.7% for white cell count, 1.2% for red cell count, 0.93% for hemoglobin, 0.78% for mean cell volume, 2.93% for platelet count, and 12.5% for reticulocytes.

### 2.4. Statistical Analysis

In the analysis of data obtained from the results of the study, SPSS 22.0 program for Windows (Chicago, IL, USA) was used. The descriptive statistics were given as mean ± standard deviation (Mean ± SD). Repeated measures analysis of variance was used to assess the effects of exercise on hematological parameters with the time serving as the within-group factor followed by *t*-tests, with the Bonferroni correction for multiple comparisons. The level of *p* < 0.05 was considered as statistically significant. Additionally, the effect of size (Cohen’s d) was calculated.

## 3. Results

Changes in hematocrit, hemoglobin, red cell parameters and platelet counts before and after acute HIIT are given in Table 1. Hematocrit levels were significantly increased immediately after exercise (*p* = 0.002, Cohen’s d = 1.84), but were significantly reduced 3 and 6 h after the exercise when compared to the pre and post values (*p* < 0.001, Cohen’s d = 2.67, and *p* < 0.001, Cohen’s d = 3.17; respectively). Hemoglobin concentrations were significantly increased immediately after the exercise (*p* < 0.001, Cohen’s d = 1.07), but were significantly reduced 3 and 6 h after the exercise when compared to the post values (*p* < 0.001, Cohen’s d = 1.61, and *p* < 0.001, Cohen’s d = 2.00; respectively). Red cell count and mean cell volume significantly increased immediately after the exercise (*p* = 0.009, Cohen’s d = 2.18, and *p* = 0.001, Cohen’s d = 0.45; respectively), but were significantly reduced 3 and 6 h after the exercise when compared to the pre and post values (*p* < 0.001, Cohen’s d = 2.88, and *p* < 0.001, Cohen’s d = 2.93; respectively and *p* < 0.001, Cohen’s d = 0.70, and *p* < 0.001, Cohen’s d = 0.62; respectively). Mean cell hemoglobin values remained unchanged in all time intervals (*p* = 0.980). Platelet counts were significantly increased immediately after the exercise (*p* = 0.001, Cohen’s d = 1.28), but were significantly reduced 3 and 6 h after the exercise when compared to the post values (*p* = 0.008, Cohen’s d = 1.11, and *p* < 0.001, Cohen’s d = 0.97; respectively). 

Total and differential white cell counts and percentages before and after acute HIIT are shown in Table 2. White cell counts were significantly increased immediately and 3 h after exercise (*p* < 0.001, Cohen’s d = 2.03), but were significantly reduced 6 h after exercise when compared to the pre and post values (*p* < 0.001, Cohen’s d = 1.00, and *p* < 0.001, Cohen’s d = 0.85; respectively). Neutrophil counts and percentages were significantly increased 3 and 6 h after exercise (*p* = 0.007, Cohen’s d = 1.68, and *p* = 0.003, Cohen’s d = 2.69; respectively), but were significantly reduced 6 h after exercise when compared to 3 h after exercise (*p* = 0.037, Cohen’s d = 0.92). Eosinophil counts were significantly increased immediately after exercise (*p* < 0.001, Cohen’s d = 0.22), but were significantly reduced 3 and 6 h after exercise when compared to the pre and post values (*p* < 0.001, Cohen’s d = 0.81, and *p* < 0.001, Cohen’s d = 0.62; respectively). However, eosinophil percentages remained unchanged in all time intervals (*p* = 0.301). Basophil counts were significantly increased immediately after exercise (*p* < 0.001, Cohen’s d = 0.57), but were significantly reduced 3 and 6 h after exercise when compared to the post values (*p* < 0.001, Cohen’s d = 1.48, and *p* < 0.001, Cohen’s d = 1.26; respectively). However, basophil percentages were lowest 3 h after exercise (*p* < 0.001) and returned to the baseline values 6 h after exercise (*p* < 0.001, Cohen’s d = 0.57). Lymphocyte counts and percentages were significantly increased immediately after exercise (*p* < 0.001, Cohen’s d = 1.17, and *p* < 0.001, Cohen’s d = 1.18; respectively), but were significantly reduced 3 and 6 h after exercise when compared to the pre and post values (*p* < 0.001, Cohen’s d = 2.35, and *p* < 0.001, Cohen’s d = 3.89; respectively). Monocyte counts were significantly reduced 3 and 6 h after exercise when compared to the post values (*p* = 0.005, Cohen’s d = 1.67 and *p* < 0.001, Cohen’s d = 1.07; respectively). However, monocyte percentages were significantly increased immediately after exercise (*p* = 0.004, Cohen’s d = 0.67), but were significantly decreased 3 h after exercise compared to the all-time intervals (*p* = 0.030).

## 4. Discussion

In this study, we investigated the responses of blood parameters to acute HIIT in the sedentary male subjects. In the present study, in general, all measured blood parameters increased immediately after exercise and returned to the baseline levels 3 or 6 h after exercise.

It has been generally shown that blood parameter values returned to resting levels 30 min after the single Wingate test or acute submaximal exercise test [20,21,22]. Current studies [17,23] showed increased blood and plasma viscosity in response to different exercise protocols. In our study, red cell counts significantly increased immediately after exercise, but these values reduced 3 and 6 h after exercise when compared to the pre and post values. In a study of Halson et al. [24], 4 weeks of intensive training significantly reduced red cell counts (10%). In contrast, Green et al. [25] did not observe any significant difference after a 4-week exercise program in the red cell counts of the sedentary subjects. In the present study, we observed a 12% increase in the hematocrit, 5.3% increase in the hemoglobin, and 8% increase in the red cell counts. This increase was similar to the previous studies.

In a recent study, Lippi et al. [26] reported a significant increase in the mean cell volume (2%) immediately after moderately long-distance half marathon running and this value returned to resting levels 3 h after exercise. Strenuous exercise is known to cause structural damage in the erythrocyte, resulting in hemolysis. Increasing the number of erythrocytes deformed by exercise can be reflected as an increase of mean cell volume in the circulation [27]. In this study, immediately after exercise, mean cell volume values were significantly increased and these values returned to resting levels 3 h after the exercise. In the present study, we observed a 1.5% increase in the mean cell volume—similar to the previous study.

In our study, platelet counts significantly increased immediately after exercise, but were significantly reduced 3 and 6 h after exercise when compared to the post values. In the present study, we observed a 31% increase in the platelet counts. Consistent with our findings, Ahmadizad et al. [22] found a significant increase in the platelet count after a single session of anaerobic exercise. It has been reported that platelet secretion increases by increasing the secretion of epinephrine during high-intensity interval training [28]. This increase was similar to the previous studies.

Heidari et al. [28] have confirmed that the white cell counts increased (more than 100%) even 30 min after single session anaerobic exercise. White blood cells are involved in all aspects of the immune system. The immune system can be activated directly via cells or the release of soluble factors. Physical activity caused changes in the distribution of the number of white blood cells [29,30]. In our study, the number of the white blood cells significantly increased immediately and 3 h after exercise, but significantly reduced 6 h after exercise when compared to the pre and post values. In the present study, we observed a 75% increase in the white cell counts. This increase was similar to the previous studies.

In the present study, after acute HIIT, an elevation was observed in all leukocyte subgroups and the values of these parameters returned to resting levels 3 h after exercise. In a review of Gabriel et al. [31], the authors noted that leukocyte subgroups such as lymphocytes percentage, basophils, eosinophils, neutrophils, lymphocytes, monocytes and monocyte percentage increased after acute anaerobic exercise. In another study, Peake et al. [32] suggested a decrease in lymphocyte values in the first hour after exercise. In the study of Close et al. [33], a 15–30% elevation in the leucocyte, neutrophil and lymphocyte counts after exercise was reported, but red blood cells remained unchanged. Moreover, in the study of Kappel et al. [34], it was found that acute exercise applied to the sedentary group caused an increase in the number of leukocytes. In the present study, we observed a 67% increase in the lymphocyte counts, and 56% increase in the monocyte counts. This increase was similar to the previous studies.

## 5. Conclusions

In conclusion, all blood parameters increased immediately after acute high-intensity interval training and their values began to return to resting levels 3 h after exercise, and completely returned to resting levels 6 h after exercise. As was seen in the current study, acute high-intensity interval exercise is considered a serious challenge to the hemorheological state in the sedentary subjects. Since some of these responses potentially could threaten subjects’ health and performance, it is recommended that subjects apply their insight about the response and adaptation of blood variables to exercise training and also further studies are needed to examine the effects of acute high-intensity interval exercise on blood parameters.

## Figures and Tables

**Table 1:** Changes in hematocrit, hemoglobin, red cell parameters and platelet counts before and after the acute high-intensity interval training (HIIT) (mean ± standard deviation (SD)) (*n* = 10).

	Pre	Post	3 h Rec	6 h Rec
Hematocrit (%)	47.43 ± 2.91	52.74 ± 2.85 ^a^	46.27 ± 1.90 ^a,b^	44.69 ± 2.18 ^a,b^
Hemoglobin (g dL^−1^)	15.75 ± 0.76	16.59 ± 0.81 ^a^	15.25 ± 0.85 ^b^	15.12 ± 0.65 ^b^
Red cell count (×10^12^ L^−1^)	5.44 ± 0.22	5.92 ± 0.22 ^a^	5.30 ± 0.21 ^b^	5.23 ± 0.25 ^a,b^
Mean cell volume (fL)	86.38 ± 2.26	87.50 ± 2.66 ^a^	85.66 ± 2.56 ^a,b^	85.96 ± 2.31 ^b^
Mean cell hemoglobin (pg)	28.71 ± 1.22	29.16 ± 1.53	28.94 ± 1.61	29.17 ± 1.05
Platelet count (×10^9^ L^−1^)	239.20 ± 46.61	314.10 ± 68.22 ^a^	248.40 ± 48.39 ^b^	257.20 ± 46.48 ^b^

^a^ *p* < 0.05 vs. statistically significant compared to the pre-exercise. ^b^ *p* < 0.05 vs. statistically significant compared to the post-exercise. Pre: at rest, Post: immediately after, 3 h Rec: 3 h after the exercise tests, 6 h Rec: 6 h after the exercise tests.

**Table 2:** Total and differential white cell counts and percentages before and after acute HIIT (mean ± SD) (*n* = 10).

	Pre	Post	3 h Rec	6 h Rec
White cell count (×10^9^ L^−1^)	7.32 ± 1.83	12.84 ± 3.37 ^a^	11.22 ± 3.58 ^a^	9.99 ± 3.30 ^a,b^
Neutrophil count (×10^9^ L^−1^)	4.02 ± 1.23	5.05 ± 1.57	8.45 ± 3.50 ^a,b^	6.64 ± 2.93 ^a,c^
Neutrophils (%)	49.83 ± 7.35	42.83 ± 6.27	72.76 ± 9.57 ^a,b^	64.52 ± 8.28 ^a,c^
Eosinophil count (×10^9^ L^−1^)	0.30 ± 0.23	0.35 ± 0.22 ^a^	0.16 ± 0.08 ^a,b^	0.19 ± 0.09 ^a,b^
Eosinophils (%)	3.66 ± 2.38	3.20 ± 2.32	1.53 ± 0.83	2.16 ± 1.26
Basophil count (×10^9^ L^−1^)	0.09 ± 0.08	0.13 ± 0.06 ^a^	0.06 ± 0.03 ^b^	0.07 ± 0.03 ^b^
Basophils (%)	1.03 ± 0.34	1.06 ± 0.46	0.58 ± 0.25 ^a,b^	0.75 ± 0.34 ^c^
Lymphocyte count (×10^9^ L^−1^)	3.11 ± 1.59	5.22 ± 1.99 ^a^	1.88 ± 0.30 ^a,b^	2.31 ± 0.41 ^a,b^
Lymphocytes (%)	36.70 ± 5.25	43.09 ± 5.65 ^a^	18.55 ± 6.90 ^a,b^	24.55 ± 6.3 ^a,b^
Monocyte count (×10^9^ L^−1^)	0.74 ± 0.37	1.16 ± 0.37	0.68 ± 0.17 ^b^	0.79 ± 0.32 ^b^
Monocytes (%)	8.79 ± 1.33	9.80 ± 1.67 ^a^	6.59 ± 2.27 ^a,b^	10.16 ± 7.59 ^c^

^a^ *p* < 0.05 vs. statistically significant compared to the pre-exercise. ^b^ *p* < 0.05 vs. statistically significant compared to the post-exercise. ^c^ *p* < 0.05 vs. statistically significant compared to 3 h after exercise. Pre: at rest, Post: immediately after, 3 h Rec: 3 h after the exercise tests, 6 h Rec: 6 h after the exercise tests.
